# Large Language Models in Medical Education: Opportunities, Challenges, and Future Directions

**DOI:** 10.2196/48291

**Published:** 2023-06-01

**Authors:** Alaa Abd-alrazaq, Rawan AlSaad, Dari Alhuwail, Arfan Ahmed, Padraig Mark Healy, Syed Latifi, Sarah Aziz, Rafat Damseh, Sadam Alabed Alrazak, Javaid Sheikh

**Affiliations:** 1 AI Center for Precision Health Weill Cornell Medicine-Qatar Doha Qatar; 2 College of Computing and Information Technology University of Doha for Science and Technology Doha Qatar; 3 Information Science Department College of Life Sciences Kuwait University Kuwait Kuwait; 4 Office of Educational Development Division of Medical Education Weill Cornell Medicine-Qatar Doha Qatar; 5 Department of Computer Science and Software Engineering United Arab Emirates University Abu Dhabi United Arab Emirates; 6 Department of Mechanical & Industrial Engineering Faculty of Applied Science and Engineering University of Toronto Toronto, ON Canada

**Keywords:** large language models, artificial intelligence, medical education, ChatGPT, GPT-4, generative AI, students, educators

## Abstract

The integration of large language models (LLMs), such as those in the Generative Pre-trained Transformers (GPT) series, into medical education has the potential to transform learning experiences for students and elevate their knowledge, skills, and competence. Drawing on a wealth of professional and academic experience, we propose that LLMs hold promise for revolutionizing medical curriculum development, teaching methodologies, personalized study plans and learning materials, student assessments, and more. However, we also critically examine the challenges that such integration might pose by addressing issues of algorithmic bias, overreliance, plagiarism, misinformation, inequity, privacy, and copyright concerns in medical education. As we navigate the shift from an information-driven educational paradigm to an artificial intelligence (AI)–driven educational paradigm, we argue that it is paramount to understand both the potential and the pitfalls of LLMs in medical education. This paper thus offers our perspective on the opportunities and challenges of using LLMs in this context. We believe that the insights gleaned from this analysis will serve as a foundation for future recommendations and best practices in the field, fostering the responsible and effective use of AI technologies in medical education.

## Introduction

We are witnessing a significant paradigm shift in the field of artificial intelligence (AI) due to the emergence of large-scale self-supervised models that can be leveraged to automate a wide variety of downstream tasks. These models are now referred to as *foundation models*, with many notable examples, such as OpenAI’s GPT-4 [1] and DALL-E [2], Meta’s SAM (Segment Anything Model) [3] and LLaMA [4], and Google’s LaMDA (Language Models for Dialog Applications) [5] and large-scale ViT (Vision Transformer) [6]. These models are trained on massive amounts of data and are capable of performing tasks related to natural language processing, computer vision, robotic manipulation, and computer-human interaction. Language-based foundation models, or large language models (LLMs), can understand and generate natural language text, allowing them to engage in human-like conversations, with coherent and contextually appropriate responses to user prompts. Remarkably, due to the advancement of these large-scale AI systems, they are now able to generate human-like content (eg, texts, images, codes, audio, and videos).

The Generative Pre-trained Transformers (GPT) series models launched by OpenAI are examples of foundation models that are based on generative AI (ie, AI models used to generate new content, such as texts, images, codes, audio, and videos, based on the training data they have been exposed to). OpenAI launched the first model of the GPT series (GPT-1) in 2018, followed by GPT-2 in 2019, GPT-3 in 2020, ChatGPT in 2022, and GPT-4 in 2023, with each iteration representing significant improvements over the previous one. GPT-4 is one of the most advanced AI-based chatbots available today. GPT-4 is an advanced multimodal foundation model that has state-of-the-art performance in generating human-like text based on user prompts [1]. Unlike previous GPT series models (eg, ChatGPT, GPT-3, and GPT-2), which accept only text inputs, GPT-4 can process image inputs, in addition to text inputs, to return textual responses [1]. Furthermore, GPT-4 has a larger model size (more parameters); has been trained on a larger amount of data; and can generate more detailed responses (more than 25,000 words), with a high level of fidelity [7]. Based on rigorous experimentation, GPT-4 capabilities demonstrate improved reasoning, creativity, safety, and alignment and the ability to process complex instructions [1]. As a result, GPT-4 is now actively used by millions of users for language translation, sentiment analysis, image captioning, text summarization, question-answering systems, named entity recognition, content moderation, text paraphrasing, personalized recommendations, text completion and prediction, programming code generation and debugging, and so forth.

Undoubtedly, the versatility and capabilities of current generative AI and LLMs (eg, GPT-4) will revolutionize various domains, with one of particular interest being medical education. The integration of such technologies into medical education offers numerous opportunities for enhancing students’ knowledge, skills, and competence. For instance, LLMs can be used to produce clinical case studies, act as virtual test subjects or virtual patients, facilitate and accelerate research outputs, develop course plans, and provide personalized feedback and assistance. However, their adoption in medical education presents serious challenges, such as plagiarism, misinformation, overreliance, inequity, privacy, and copyright issues. In order to shift medical education practices from being information-driven to being AI-driven through the use of LLMs, it is essential to acknowledge and address the concerns and challenges associated with the adoption of LLMs. This is necessary to ensure that students and educators understand how to use these tools effectively and appropriately to fully leverage their potential. To this end, the objective of this paper is to explore the opportunities, challenges, and future directions of using LLMs in medical education. This paper uses GPT-4 as a case study to discuss these opportunities and challenges, as it is a state-of-the-art generative LLM that was available at the time of writing.

## Opportunities

### Overview

**Figure 1:**
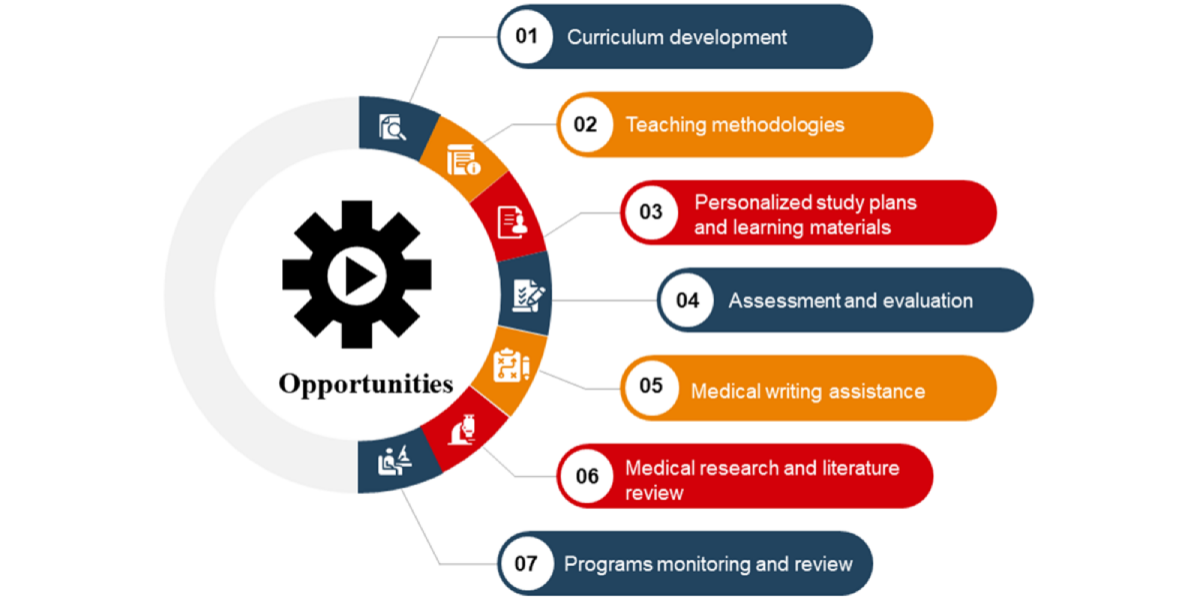
Opportunities of large language models in medical education.

LLMs have the potential to significantly impact all phases of medical education programs, offering numerous benefits in various aspects, including curriculum planning, delivery, assessments, programmatic enhancements, and research [8-30]. This section elucidates and illustrates the specific opportunities and applications of LLMs that can be leveraged to deliver a more efficient, effective, personalized, and engaging medical education system that is better equipped to prepare future health care professionals. Figure 1 shows the main opportunities of LLMs in medical education.

### Curriculum Development

Medical curriculum planning is a complex process that requires careful consideration of various factors, including educational objectives, teaching methodologies, assessment strategies, and resource allocation [31]. LLMs, like GPT-4, can play a significant role in enhancing this process by conducting needs assessments and analyses and providing expert-level knowledge and insights on various medical topics, helping educators identify content gaps and ensure comprehensive coverage of essential subjects [8,17]. Additionally, GPT-4 can assist in developing measurable learning objectives for each phase of a medical program curriculum and customizing it to meet the diverse needs of individual learners, fostering personalized and adaptive learning experiences. By analyzing students' performance data, LLMs can suggest targeted interventions and recommend specific resources to address learning gaps and optimize educational outcomes [16,17]. Furthermore, this integration of LLMs and GPT-4 into medical curriculum planning can support faculty in designing, updating, or modifying a medical curriculum; LLMs can provide suggestions for course content, learning objectives, and teaching methodologies based on the emerging trends and best practices in medical education, freeing up more time for faculty to focus on other teaching aspects [32-34].

### Teaching Methodologies

LLMs, like GPT-4, can be used to augment existing teaching methodologies in medical education programs, enhancing the overall learning experience for students. For example, LLMs can supplement lecture content by providing real-time clarifications, additional resources, and context to complex topics, ensuring a deeper understanding for students [35,36]. For small-group sessions, GPT-4 can facilitate discussions by generating thought-provoking questions, encouraging peer-to-peer interactions, and fostering an engaging and collaborative learning environment. For virtual patient simulations, LLMs can create realistic virtual patient scenarios, ask questions, interpret responses, and provide feedback, allowing students to practice clinical reasoning, decision-making, and communication skills in a safe and controlled setting. For interactive medical case studies, GPT-4 can generate case studies that are tailored to specific learning objectives and guide students through the diagnostic process, treatment options, and ethical considerations, thereby allowing students to interactively explore both common conditions and rare conditions, which can help to prepare them for real-world clinical practice [37]. An example of using ChatGPT (GPT-4) to create interactive case studies for medical students is included in Multimedia Appendix 1. For clinical rotations, as virtual mentors, LLMs can help students apply theoretical knowledge to real-world situations by offering instant feedback and personalized guidance to reinforce learning and address misconceptions.

### Personalized Study Plans and Learning Materials

By leveraging the power of LLMs and generative AI tools, students can input information about their individual strengths, weaknesses, goals, and preferences to generate study plans that are tailored to their specific needs. This level of personalization ensures that each student's unique learning style and pace are taken into account, leading to more efficient and effective learning [38]. Moreover, LLMs, like GPT-4, can also generate personalized learning materials, including concise summaries, flash cards, and practice questions, that target specific areas where a student needs improvement. An example of using an LLM, like ChatGPT, to provide personalized explanations of medical terminology (ie, *aphthous stomatitis*) to students at different levels (premedical students, year 2 medical students, and year 4 medical students) is presented in Multimedia Appendix 2. Tailored resources can help students focus on the most relevant content, optimizing their study time and enhancing knowledge retention. Furthermore, an iterative feedback loop could be established wherein students use LLM-generated materials and provide feedback, which is then used to fine-tune the LLM's outputs. Over time, this could lead to increasingly accurate and effective personalized learning materials.

### Assessment and Evaluation

LLMs and GPT-4 can play a significant role in designing comprehensive assessment plans and enhancing the evaluation process in medical education [14,18,26]. They can be utilized to (1) develop comprehensive, well-rounded assessment plans that incorporate formative and summative evaluations, competency-based assessments, and effective feedback mechanisms; (2) align assessment methods with learning objectives by analyzing learning objectives and suggesting appropriate assessment methods that accurately measure students' progress toward achieving the desired competencies; and (3) provide prompt feedback and rubrics by automating the process of providing timely and actionable feedback to students, identifying areas of strength and weakness, and offering targeted suggestions for improvement. Additionally, GPT-4 can assist in the creation of transparent and consistent grading rubrics, ensuring that students understand the expectations and criteria for success.

### Medical Writing Assistance

LLMs have become valuable tools in medical writing, offering a range of benefits to medical students and medical researchers [37,39-43]. LLMs, like GPT-4, can assist medical students and educators in selecting appropriate language, terminology, and phrases for use in their writing, ensuring accuracy and readability for their intended audience. Furthermore, LLMs can provide guidance on writing style and formatting, helping students to improve the clarity and coherence of their work. By leveraging these chatbots' capabilities, medical students can streamline their writing process and produce high-quality work, resulting in time that can be reallocated to other aspects of their studies.

### Medical Research and Literature Review

LLMs are valuable tools for medical research and literature reviews, providing a faster, more efficient, and more accurate means of gathering and analyzing data [26,28,44-46]. With the ability to access, extract, and summarize relevant information from scientific literature, electronic medical records, and other sources, these chatbots enable medical students and researchers to quickly and efficiently gather the information they need for their reports, papers, and research articles. By leveraging the data extraction capabilities of LLMs, medical students and researchers can more easily access and analyze the vast amounts of information available to them (Multimedia Appendix 3). This ensures that their research is grounded in accurate and reliable data, allows them to make well-informed conclusions based on their findings, and frees up valuable time and resources that can be directed toward other important aspects of the research process. Moreover, when writing research papers, medical students can use LLMs for help with generating outlines and drafting introductions or conclusions; LLMs can also suggest possible ways to discuss and analyze results (Multimedia Appendix 4).

### Program Monitoring and Review

LLMs and generative AI tools, when integrated into curriculum management systems, have enormous potential to transform the monitoring and review of medical education programs. By analyzing data collected through various sources, including student feedback, testing results, and program delivery data, LLMs like GPT-4 can provide program leaders with valuable insights into the efficacy of their programs. LLMs can identify areas of improvement, monitor trends in student performance, and provide benchmarks against which program performance can be evaluated. LLMs can also analyze national health priorities and community needs to help programs adapt and adjust their objectives and allocation of resources accordingly. By leveraging these tools, program leaders can gain insights and make data-driven decisions that enhance the quality and effectiveness of medical education programs.

## Challenges

### Overview

**Figure 2:**
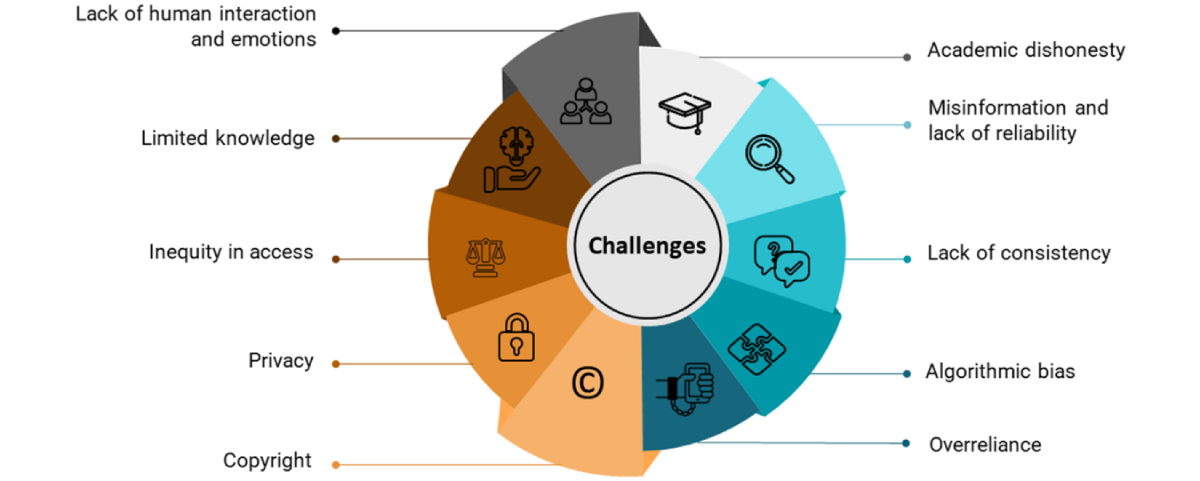
Challenges of large language models in medical education.

Despite the abovementioned opportunities that LLMs and generative AI tools can provide, they have limitations in medical education. These challenges and limitations are discussed in the following subsections. Figure 2 shows the main challenges of LLMs in medical education.

### Academic Dishonesty

The ability of LLMs to respond to short-answer and multiple-choice exam questions can be exploited for cheating purposes [47]. As mentioned earlier, LLMs can write medical essays that are difficult to distinguish from human-generated essays, which may increase plagiarism. Although several tools (eg, GPTZero, Originality.AI, OpenAI AI Text Classifier, and Turnitin AI Writing Detector) have been developed to detect AI-generated text, students may still be able to make their AI-generated essays undetectable to such tools. Specifically, a study demonstrated that adding 1 word (“amazing”) to an AI-generated text reduced the fake level (ie, generated by AI) detected by a tool from 99% to 24% [48]. Although this is just 1 example, it still increases and highlights apprehensions regarding the effectiveness of such tools in detecting and preventing plagiarism.

### Misinformation and Lack of Reliability

Although recent LLMs (eg, GPT-4) have significantly reduced hallucinations in comparison with earlier models [1], due to inaccurate training data, recent LLMs still generate incorrect or inaccurate information that is convincingly written. Given the authoritative writing style generated by these systems, students may find it challenging to differentiate between genuine knowledge and unverified information. As a result, they may not scrutinize the validity of information and end up believing inaccurate or deceptive information [49]. Further, such misinformation may make LLMs untrustworthy among users and thus may decrease the adoption of LLMs. As an example of misinformation, studies showed that LLMs, such as GPT-4, either include citations that do not exist in generated articles or include citations that are irrelevant to the topic [41,50-52]. This raises the question of how to guarantee that generative AI tools and LLMs remain assistive technologies and not propagators of false or misleading health information.

### Lack of Consistency

Recent LLMs and generative AI tools generate different outputs for the same prompt. Although this feature may be helpful in some cases, it has several disadvantages [53]. First, generating different responses to the same prompt may prevent educators from detecting whether the text was generated by AI. Second, this feature may produce contradicting responses on the same topic. Finally, this feature may generate responses with different qualities. For example, in a study [48], 3 researchers at the same location asked an LLM-based chatbot the exact same question at the same time, but they received 3 different responses of different quality. Specifically, the first researcher received a more up-to-date, complete, and organized response compared to the responses that the second and third researchers received [48]. Accordingly, one may inquire about the methods to guarantee fair access, for all users (students and educators), to identical, up-to-date, and high-quality learning materials.

### Algorithmic Bias

Given that recent LLMs (eg, GPT-4) are trained on a large corpus of text data from the internet (eg, websites, books, news articles, scientific papers, and movie subtitles), it is likely that they are trained on biased or unrepresentative data. OpenAI has acknowledged that GPT-4 may still generate biased responses like earlier GPT models, thereby reinforcing social biases and stereotypes [1]. For example, if an LLM was trained on data related to disease among a certain ethnic group, then it is likely that it generates responses (eg, essays, exams, and clinical case scenarios) that are biased toward that group. According to a study [54], an LLM that was trained on a vast corpus of internet text demonstrated gender bias in its output.

### Overreliance

As mentioned earlier, recent generative AI tools (eg, GPT-4) have a tendency to make up facts and present incorrect information in more convincing and believable ways [1]. This may cause users to excessively trust generative AI tools, thereby increasing the risk of overreliance. Therefore, the use of generative AI tools may hinder the development of new skills or even lead to the loss of skills that are foundational to medical student development, such as critical thinking, problem-solving, and communication. In other words, the ease with which generative AI tools can provide answers could lead to a decrease in students’ motivation to conduct independent investigations and arrive at their own conclusions or solutions. This raises the question of how generative AI tools can be used to improve rather than reduce critical thinking and problem-solving in students.

### Lack of Human Interaction and Emotions

Current LLMs are unable to deliver the same degree of human interaction as an actual educator or tutor. This is because, at present, (1) their capabilities are restricted to a textual interface, (2) they are incapable of recognizing the physical gestures or movements of students and educators, and (3) they cannot reveal any emotions. The absence of human interaction can negatively affect students who prefer a personal connection with their educator. According to a study conducted by D'Mello and colleagues [55], students who engaged with a virtual tutor that imitated human-like emotional behavior demonstrated superior learning outcomes compared to those who engaged with a virtual tutor that lacked such behavior. Hence, it is worth considering ways to humanize generative AI tools not just in their ability to think and provide responses but also in terms of exhibiting emotions and possessing a distinctive personality.

### Limited Knowledge

LLMs, like GPT-4, depend on the data used for training, which cover a wide range of general information but might not always encompass the latest or most specialized medical knowledge. This constraint impacts the reliability and precision of the information generated by LLMs in medical education environments, where accuracy and expertise are essential [26]. Moreover, the knowledge base of most LLMs is presently static, which means that they cannot learn and adjust in real time as new medical information emerges. However, the field of medicine is constantly evolving, with novel research findings, guidelines, and treatment protocols being regularly introduced [56]. Additionally, the restricted knowledge of current LLMs in medical education could result in a superficial understanding of complex medical concepts, lacking the necessary depth and context for effective learning. For instance, while GPT-4 can produce text that seems coherent and factually correct at first glance, it may not always capture the subtleties and complexities of medical knowledge, thus falling short in providing comprehensive and accurate guidance for medical students and educators.

### Inequity in Access

Generative AI tools and LLMs may increase the inequity among students and educators, given that these tools are not equally accessible to all of them. For example, although most generative AI tools can communicate in several languages, in addition to English, and outperform earlier chatbots in this aspect, their proficiency in each language varies based on the amount and quality of training data available for each language [1]; thus, students and educators who are not proficient in English are less likely to use them. Further, generative AI tools may be less accessible to (1) those who are not familiar with using technologies or AI tools; (2) those who do not have access to the necessary technology (eg, internet and computers); (3) those who cannot afford subscription fees (eg, US $20/month for GPT-4); and (4) those with disabilities, such as blindness or motor impairment.

### Privacy

When communicating with LLMs, students and educators may reveal their personal information (eg, name, email, phone number, prompts, uploaded images, and generated images). OpenAI acknowledges that it may use users’ personal information for several purposes, such as analyzing, maintaining, and improving its services; conducting research; preventing fraud, criminal activity, or misuse of its services; and complying with legal obligations and legal processes [57]. Moreover, OpenAI may share users’ personal information with third parties without further notice to users or users’ consent [57]. A recent reflection of these concerns is Italy's data protection group discontinuing access to ChatGPT while it conducts an investigation around data use and collection practices, in alignment with requirements of the General Data Protection Regulation [58]. In addition, LLM use during clerkship clinical rotations for patient care (eg, SOAP [Subjective, Objective, Assessment, and Plan] note generation) could result in unintended patient privacy breaches. Questions surrounding how to safeguard student and patient data should be central in curricular discussions.

### Copyright

LLMs may be trained on copyrighted materials (eg, books, scientific articles, and images), thereby potentially producing text that bears similarity to or even directly copies content protected by copyright, which could potentially impact downstream uses. Such a situation brings up apprehensions regarding the utilization of content created by generative AI tools (eg, educational materials, presentations, course syllabi, quizzes, and scientific papers) without appropriate acknowledgment and authorization from the copyright holder. There are ongoing discussions related to authorship rights for articles that are written by using LLMs. Although various publishers and editors do not accept listing such tools as coauthors (eg, those of *Nature*, *Jinan Journal*, and *eLife*), others do (eg, those of *Oncoscience* [59], *Nurse Education in Practice* [60], and medRxiv [61]). As this is an area likely to evolve, it raises questions regarding how students and educators should acknowledge the use of these systems while complying with professional and regulatory expectations.

## Future Directions

### Overview

Considering the opportunities and challenges presented by the use of LLMs and generative AI tools in medical education, we discuss future directions, targeting academic institutions, educators, students, developers, and researchers. We argue that those who embrace the use of the technology, including LLMs, will challenge the status quo and will likely be better positioned and higher performing than those who do not. Therefore, the following recommendations and future directions can be useful to all of the previously mentioned stakeholders and many others.

### Academic Institutions

With the rise of generative AI tools and LLMs, there is a fear that in the future, these technologies may make the human brain dormant in nearly all tasks, including some of the basic ones. Now more than ever, medical schools and academic institutions need to consider the appropriate strategies to incorporate the use of LLMs into medical education. One possibility is to develop guidelines or best practices for the use of AI tools in their assignments. These guidelines should explain to students how to properly disclose or cite any content generated by LLMs when writing essays, research papers, and assignments. Academic institutions may also subscribe to tools that can detect AI-generated text, such as Turnitin, ZeroGPT, and Originality.AI. Academic institutions should provide training sessions and workshops to teach students and educators how to effectively and ethically use such tools in medical education. Ultimately, academic institutions should favor student-centered pedagogy that nurtures building trusting relationships that focus on *assessment for learning* and do not entirely focus on *assessment of learning* [62].

### Educators

Given the rapid, explosive advances driven by the expected use GPT-4 and other LLMs, medical educators are encouraged to embrace these technologies rather than stay away from them. With AI’s rapid evolution, it is paramount for medical educators to upskill their competencies in utilizing generative AI tools effectively within medical curricula. Current medical curricula do not include education on the proper use of AI. Content covering such technologies and their application to medicine (eg, disease discovery) should be included. Medical educators should consider how LLMs can be integrated into medical education, thus requiring them to reconsider the teaching and learning process. This can be done through updating course syllabi to set and clarify the objectives of the use of LLMs (eg, GPT-4), as well as by reflecting on their use in practice and their impact on the profession.

Assignments will also have to be reconsidered, and educators should strive to assign multimodal activities that require high-order thinking, creativity, and teamwork. For example, educators could use oral exams and presentations, hands-on activities, and group projects to assess their students’ analytical and critical reasoning, the soundness and precision of their arguments, and their persuasive capabilities. Educators may consider involving students in peer evaluations and exercise “teach-back.”

Because health care is complex and often involves high stakes, it is paramount that educators also explain to their medical students the abovementioned limitations of LLMs. For example, educators should highlight the importance of proper citation and attribution in medical school, as well as how to avoid potential user privacy and copyright issues, misinformation, and biases. We recommend that educators discourage reliance that can lead to reduced clinical reasoning skills. Instead, educators should encourage students to check, critique, and improve responses generated by LLMs. Educators should emphasize that these technological tools should be continually monitored by human experts and that they should be used with guidance and critical thinking before acting on any of their recommendations.

Although LLMs, like GPT-4, are powerful tools capable of generating detailed, personalized study plans and learning materials, they are not infallible. They are as good as the data that they have been trained on, and there is always a risk of inaccuracies or misinterpretations, particularly when dealing with complex, nuanced fields, such as medical education. Therefore, we believe that it is crucial to incorporate human input or expert-reviewed content into the process of developing such tools. For instance, subject matter experts, such as experienced medical educators or practitioners, could review and validate the content generated by an LLM. They could provide the correct context, ensure that the material aligns with current medical standards and guidelines, and verify the content's relevance to students’ specific learning needs.

### Students

Students should ethically and safely use these tools and technologies in a constructive manner to thrive outside of the classroom, in a world that is rapidly being dominated by AI. Similar to educators, medical students also need to elevate their skills and competencies in effectively leveraging and utilizing generative AI tools and LLMs in their practices. It is paramount that students acknowledge the use of LLMs in their medical and academic work and, at the same time, do so ethically and responsibly.

### Developers

Developers of generative AI tools bear the responsibility of meticulously developing generative AI tools while taking into account prevalent constraints, such as inequality, privacy, impartiality, contextual understanding, human engagement, and misinformation. Although recent generative AI technologies, like GPT-4 and ChatGPT, possess the ability to communicate in various languages, their performance is notably more effective in English compared to their performance in other languages. This could be attributed to the lack of data sets and corpora in languages other than English (eg, Arabic) [63]. Developers and researchers should collaborate to build large data sets and corpora in other languages to improve the performance of LLMs when using such languages [63,64]. To tackle the challenges of fairness and equity, developers need to create generative AI technologies that can accommodate the varied requirements and backgrounds of users, particularly for underprivileged or marginalized students and educators. For example, developers ought to equip generative AI tools with the capability to interact with students and educators through voice, visuals, and videos, as well as text, to make them more humanized and accessible to those with disabilities (eg, blindness).

With some generative AI tools creating or “faking” certain articles or information, it is essential for developers to clearly state and discern facts from fiction in the outputs. Additionally, developers should also make an effort to develop more humanized LLMs that consider the virtual relationship that has been developed between humans and machines. The development of generative AI tools should rely on various theories that consider relationship formation among humans, such as social exchange theory. When developing generative AI technologies, it is also essential to adhere to user-focused design principles while taking into account the social, emotional, cognitive, and pedagogical dimensions [65]. We recommend that developers create responsible generative AI tools that correspond with core human principles and comply with our legal system.

Developers play a crucial role in integrating ChatGPT into medical education platforms, drawing inspiration from its use in popular educational platforms, such as Duolingo and Khan Academy. By examining these examples, they can design and develop innovative learning experiences for medical students who use LLMs. Duolingo and Khan Academy use ChatGPT to provide personalized learning experiences based on the individual needs and progress of each student. This approach can be adopted in medical education to create tailored study plans and learning materials that cater to the unique strengths, weaknesses, and learning styles of medical students. Both Duolingo and Khan Academy use ChatGPT to offer real-time feedback and guidance to learners as they engage with the platform. In the context of medical education, ChatGPT could be integrated into learning management systems or virtual patient simulations to provide instant feedback on students' performance, diagnostic decisions, or treatment plans. By giving students immediate access to targeted guidance and correction, ChatGPT can facilitate continuous improvement and foster a deeper understanding of medical principles. Duolingo utilizes ChatGPT to create interactive, conversation-based lessons that help learners practice their language skills in a more engaging and natural manner. Similarly, ChatGPT can be used in medical education to develop interactive learning modules that allow students to practice clinical communication skills, such as taking patient histories, explaining diagnoses, or discussing treatment options. Khan Academy leverages ChatGPT to facilitate peer-to-peer interactions and support, enabling students to learn from each other and collaborate on problem-solving tasks. In medical education, ChatGPT could be used to create virtual study groups, in which students can discuss clinical cases, share insights, and work together to solve complex medical problems.

### Researchers

There is an urgent need to conduct more empirical and evidence-based human-computer interaction and user interface design research for the use of LLMs in medical education. Researchers should explore ways to strike a balance between using these technologies and maintaining the essential human interaction and feedback in education to enhance learning and teaching experiences and outcomes [48]. Further, research is required to investigate the impact of LLMs on students’ learning processes and outcomes. Lastly, there is a need to delve deeper into the possible consequences of overdependence on LLMs in medical education [48].

## Conclusion

In conclusion, LLMs are double-edged swords. Specifically, LLMs have the potential to revolutionize medical education, enhance the learning experience, and improve the overall quality of medical education by offering a wide range of applications, such as acting as a virtual patient and medical tutor, generating medical case studies, and developing personalized study plans. However, LLMs do not come without challenges. Academic dishonesty, misinformation, privacy concerns, copyright issues, overreliance on AI, algorithmic bias, lack of consistency and human interaction, and inequity in access are some of the major hurdles that need to be addressed.

To overcome these challenges, a collaborative effort is required from educators, students, academic institutions, researchers, and developers of generative AI tools and LLMs. Rather than banning them, medical schools and academic institutions should embrace generative AI tools and develop clear guidelines and rules for the use of these technologies for academic activities. Institutional efforts may be required to help students and educators develop the skills necessary to incorporate the ethical use of AI into medical training. Educators should use new teaching philosophies and redesign assessments and assignments to allow students to use such technologies. Students should ethically and safely use these technologies in a constructive manner. Developers have a duty to carefully design such technologies while considering common limitations, such as inequity, privacy, unbiased responses, lack of context and human interaction, and misinformation.

## Appendix

Multimedia Appendix 1 Example of using ChatGPT (GPT-4) to create interactive case studies for medical students.

Multimedia Appendix 2 Example of using ChatGPT (GPT-4) to provide personalized explanations of medical terminology to students at different levels.

Multimedia Appendix 3 Example of using large language models (Petal) for document analysis.

Multimedia Appendix 4 Example of using ChatGPT (GPT-4) to provide an outline and references for research papers.

## Abbreviations

AI: artificial intelligence
GPT: Generative Pre-trained Transformers
LaMDA: Language Models for Dialog Applications
LLM: large language model
SAM: Segment Anything Model
SOAP: Subjective, Objective, Assessment, and Plan
ViT: Vision Transformer
