# Supplementary material for: Large Language Models in Medical Education: Opportunities, Challenges, and Future Directions
Source: JMIR Med Educ. 2023 Jun 1;9:e48291. doi: 10.2196/48291 (PMC10273039; doi:10.2196/48291)
Supplement: Multimedia Appendix 1 [file mededu_v9i1e48291_app1.docx]

Multimedia Appendix 1: Example of using ChatGPT (GPT-4) to create interactive case studies for medical students.


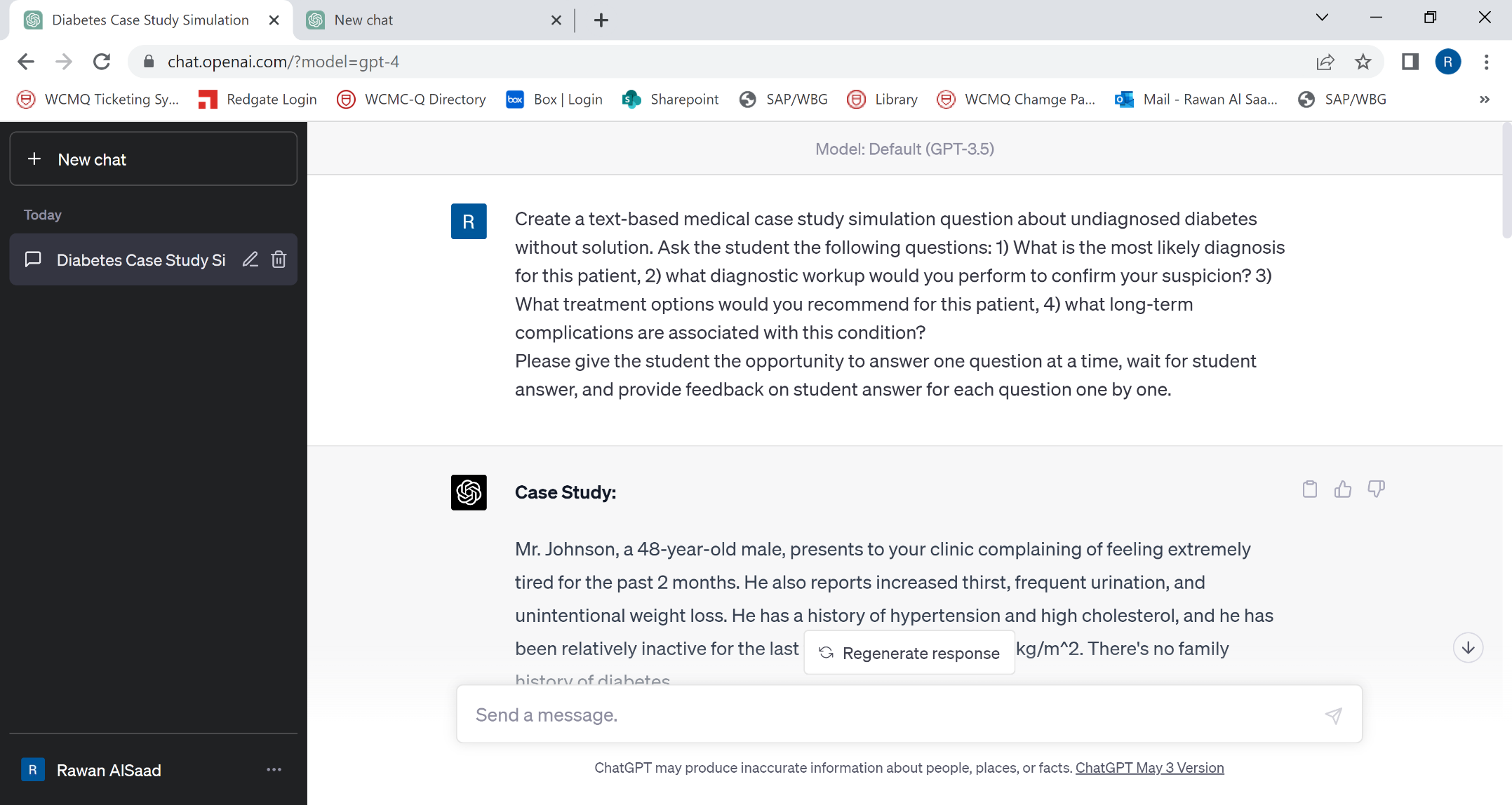


Figure S1: Case study user prompt


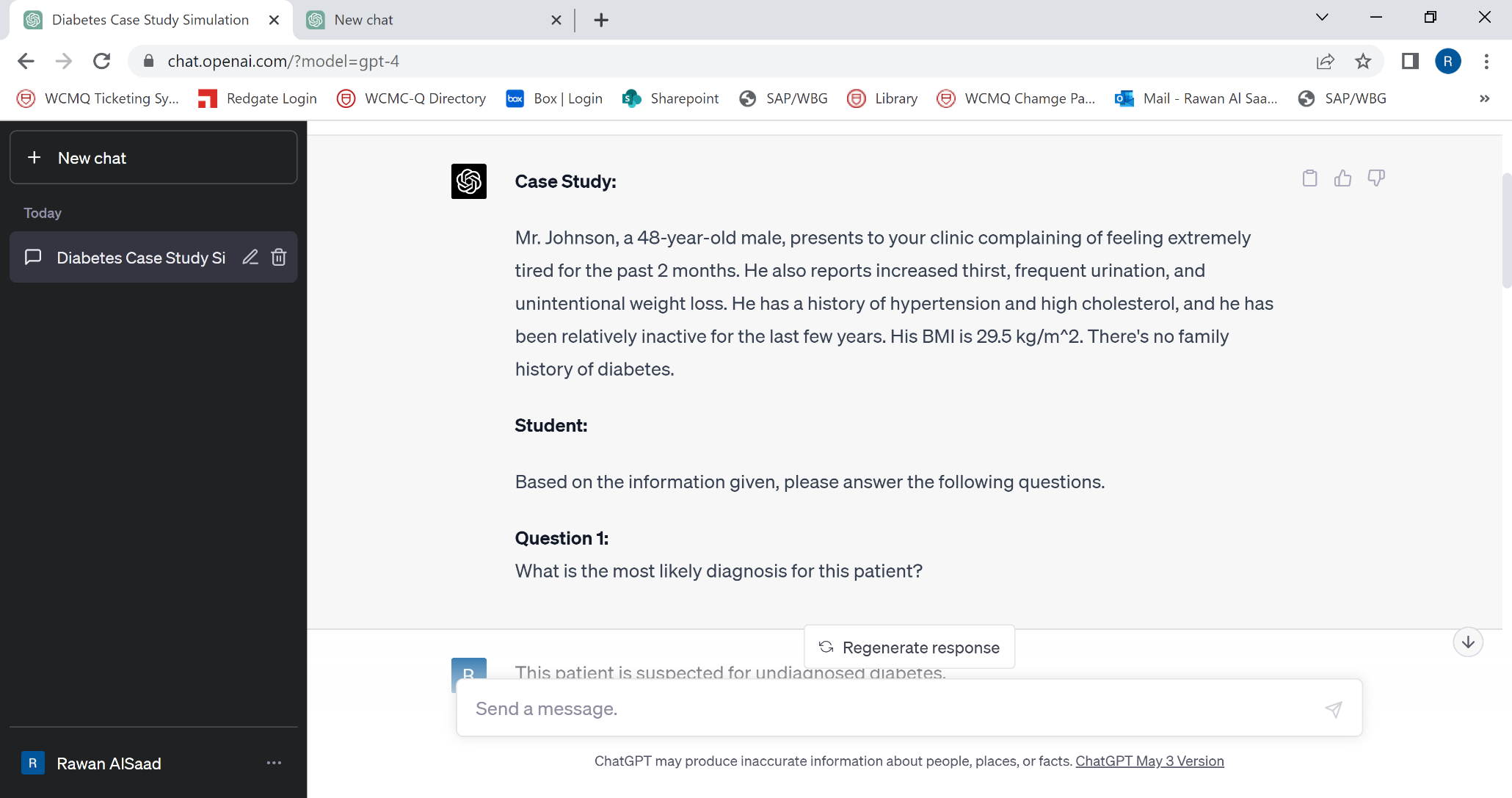


Figure S2: GPT-4 response. It creates a new case study with every new prompt.

Figure S3: Question 1. Student answer and GPT-4 feedback.


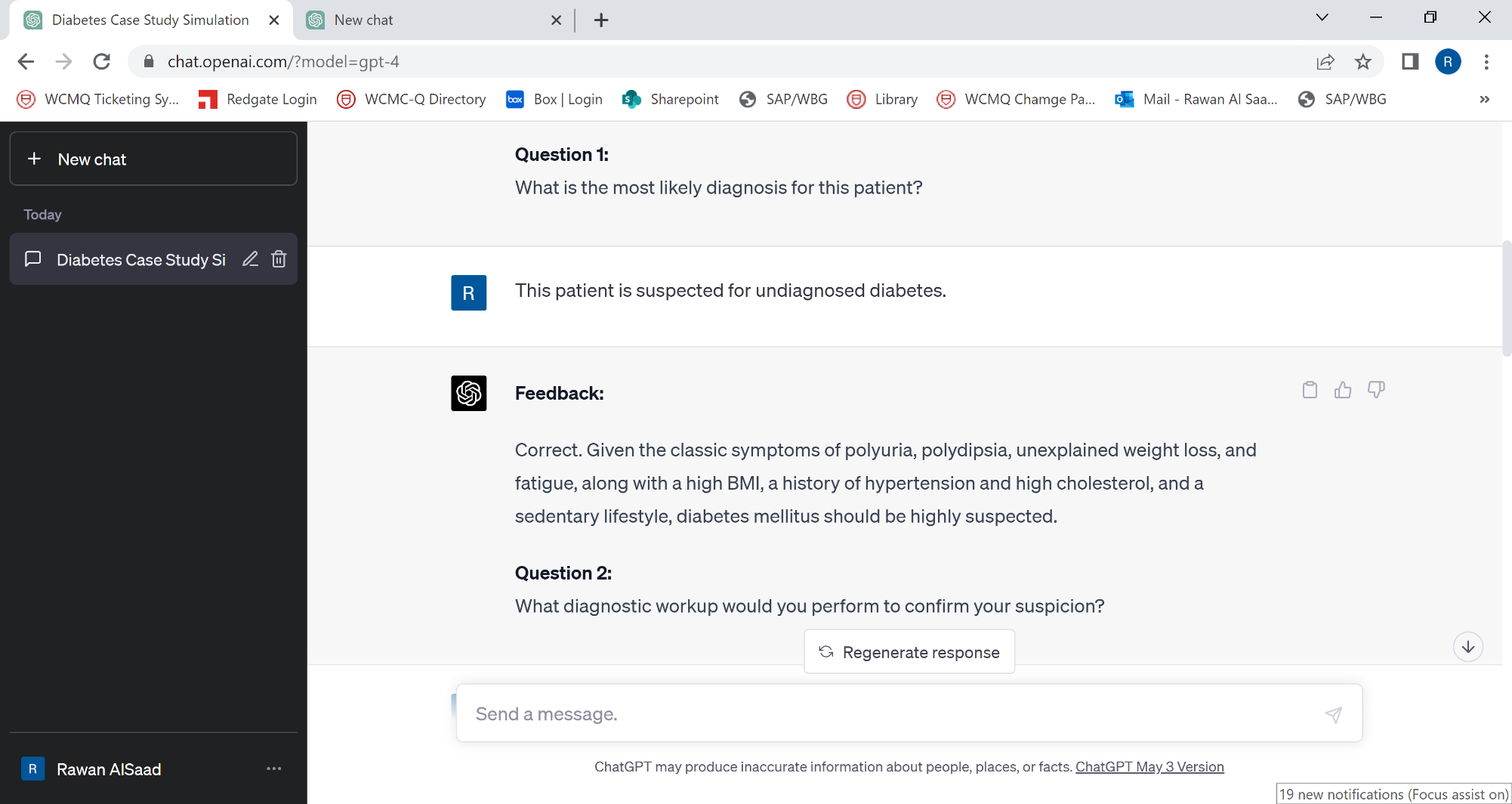


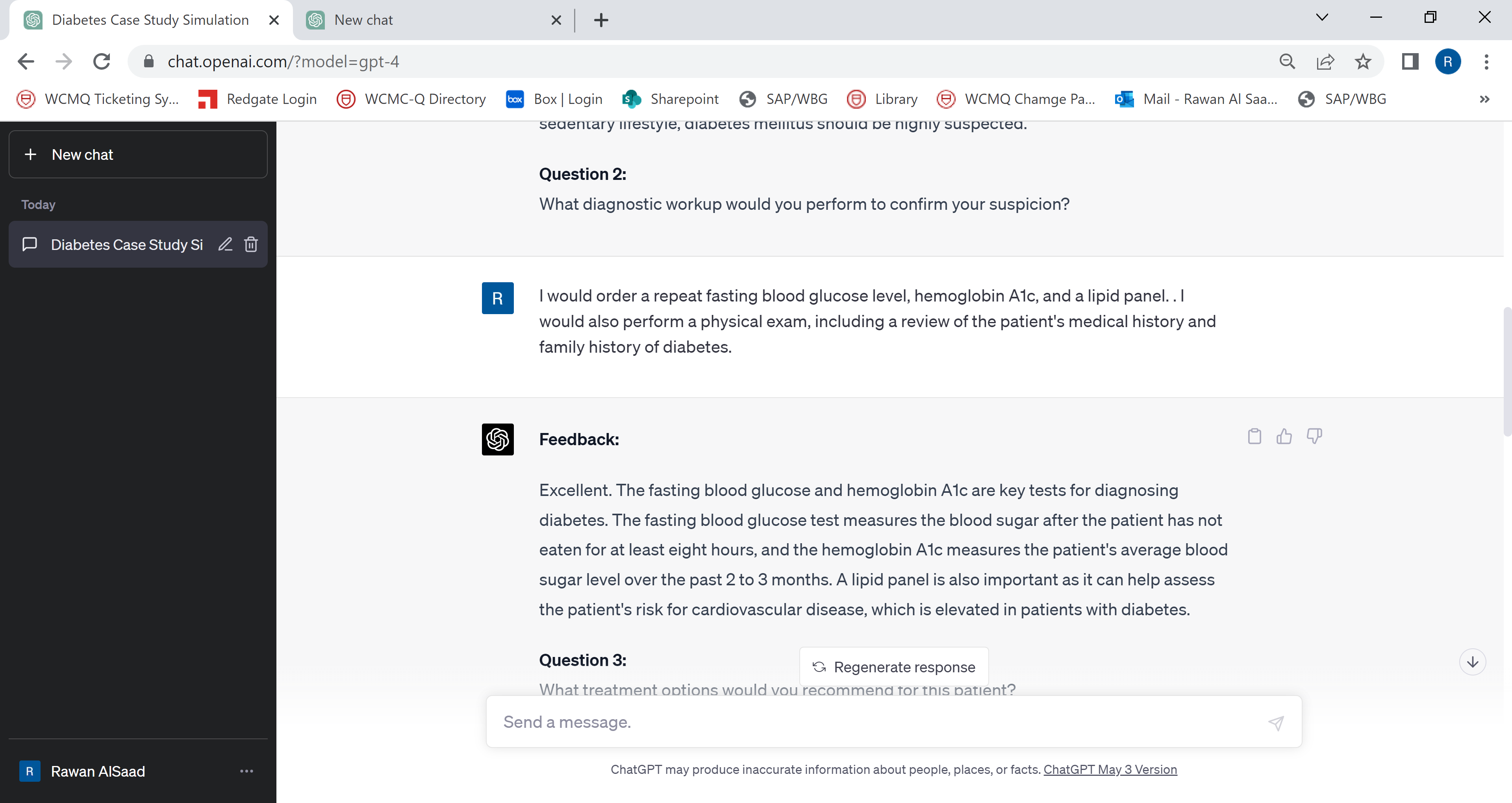


Figure S4: Question 2. Student answer and GPT-4 feedback.


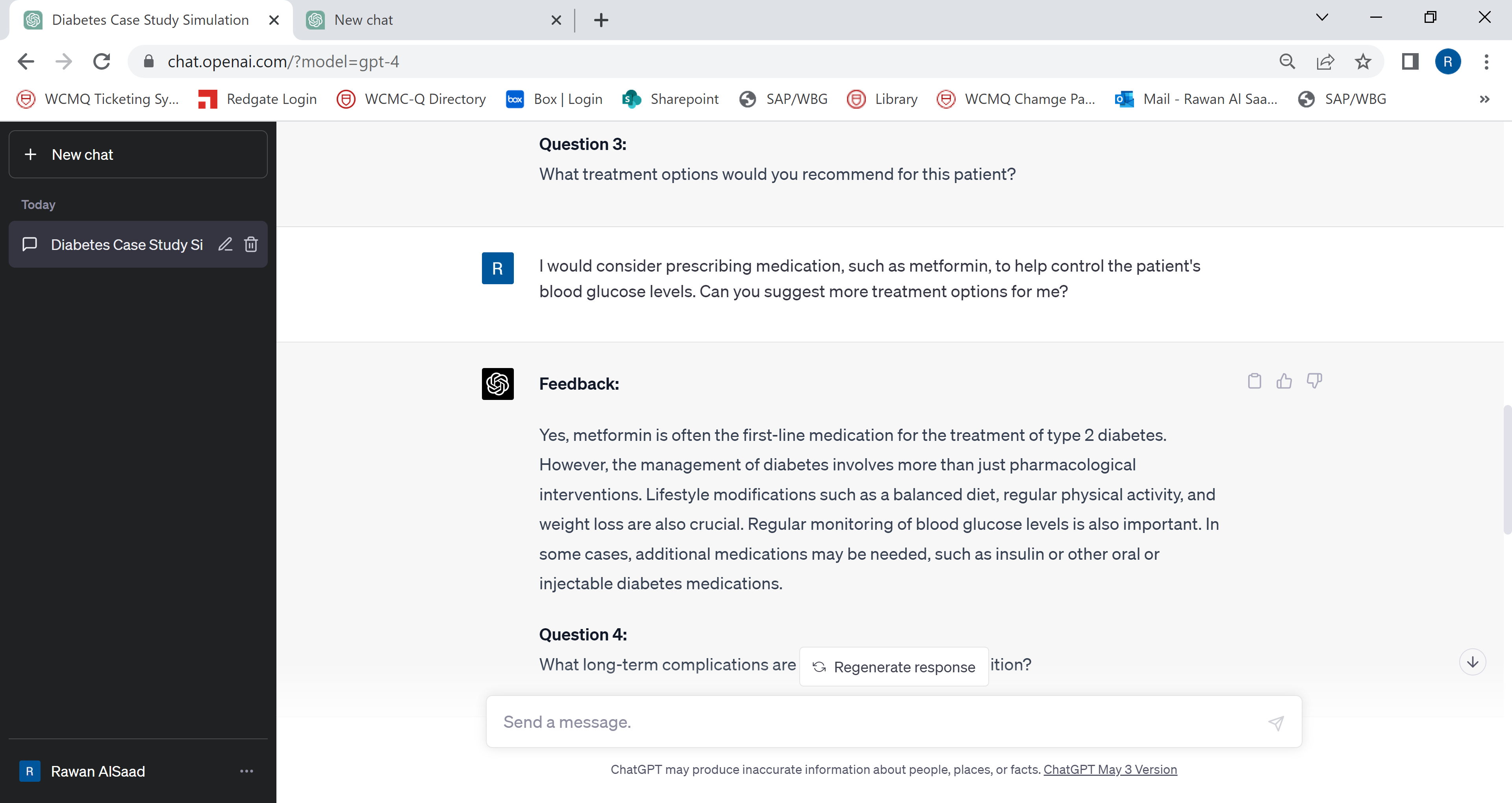


Figure S5: Question 3. Student answer and GPT-4 feedback.


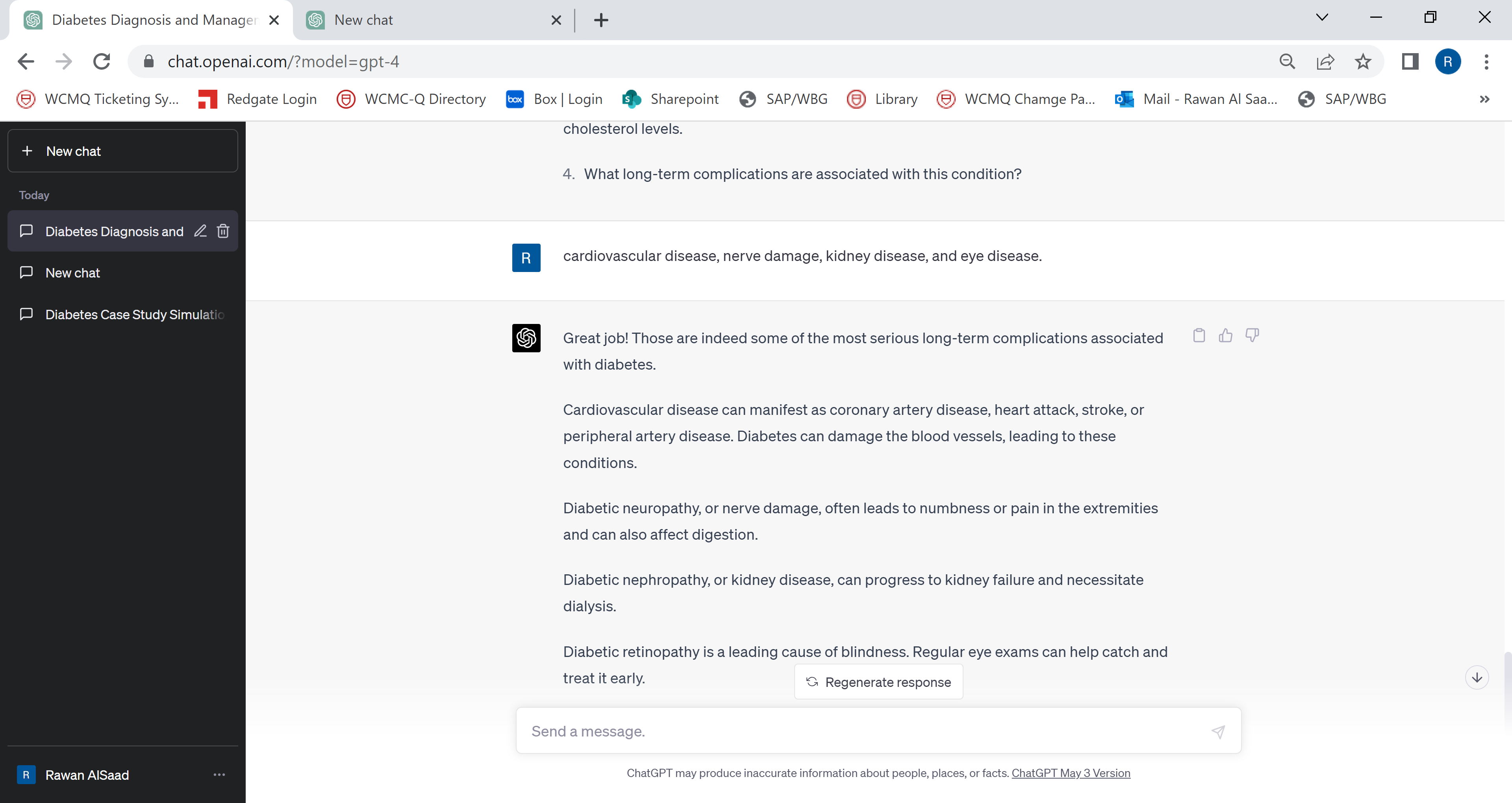


Figure S6: Question 4. Student answer and GPT-4 feedback.
