# Supplementary material for: Large Language Models in Medical Education: Opportunities, Challenges, and Future Directions
Source: JMIR Med Educ. 2023 Jun 1;9:e48291. doi: 10.2196/48291 (PMC10273039; doi:10.2196/48291)
Supplement: Multimedia Appendix 2 [file mededu_v9i1e48291_app2.docx]

Multimedia Appendix 2: Example of using ChatGPT (GPT-4) to provide personalized explanations of medical terminology to students at different levels

Figure S1: User prompt to GPT-4.


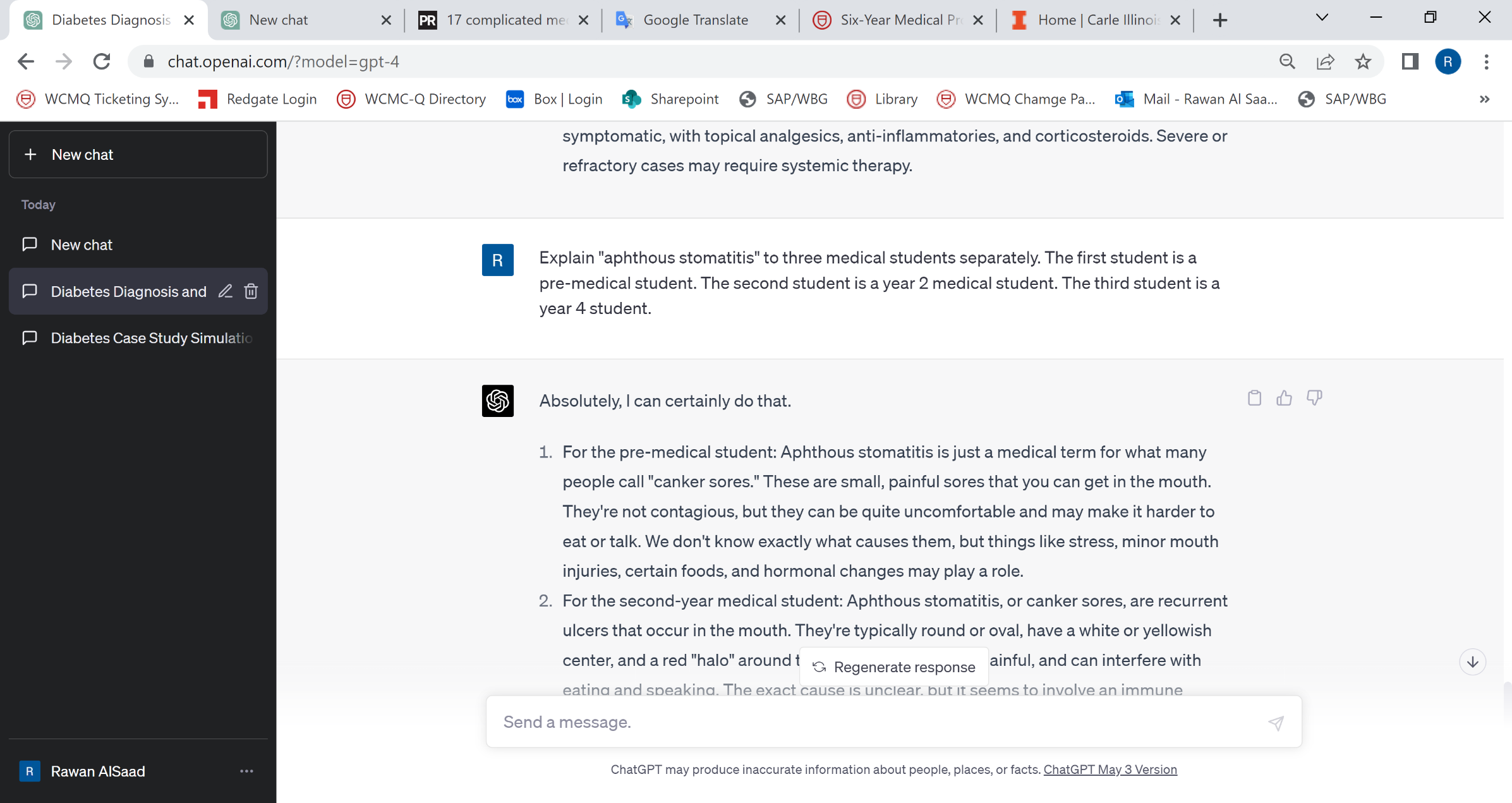


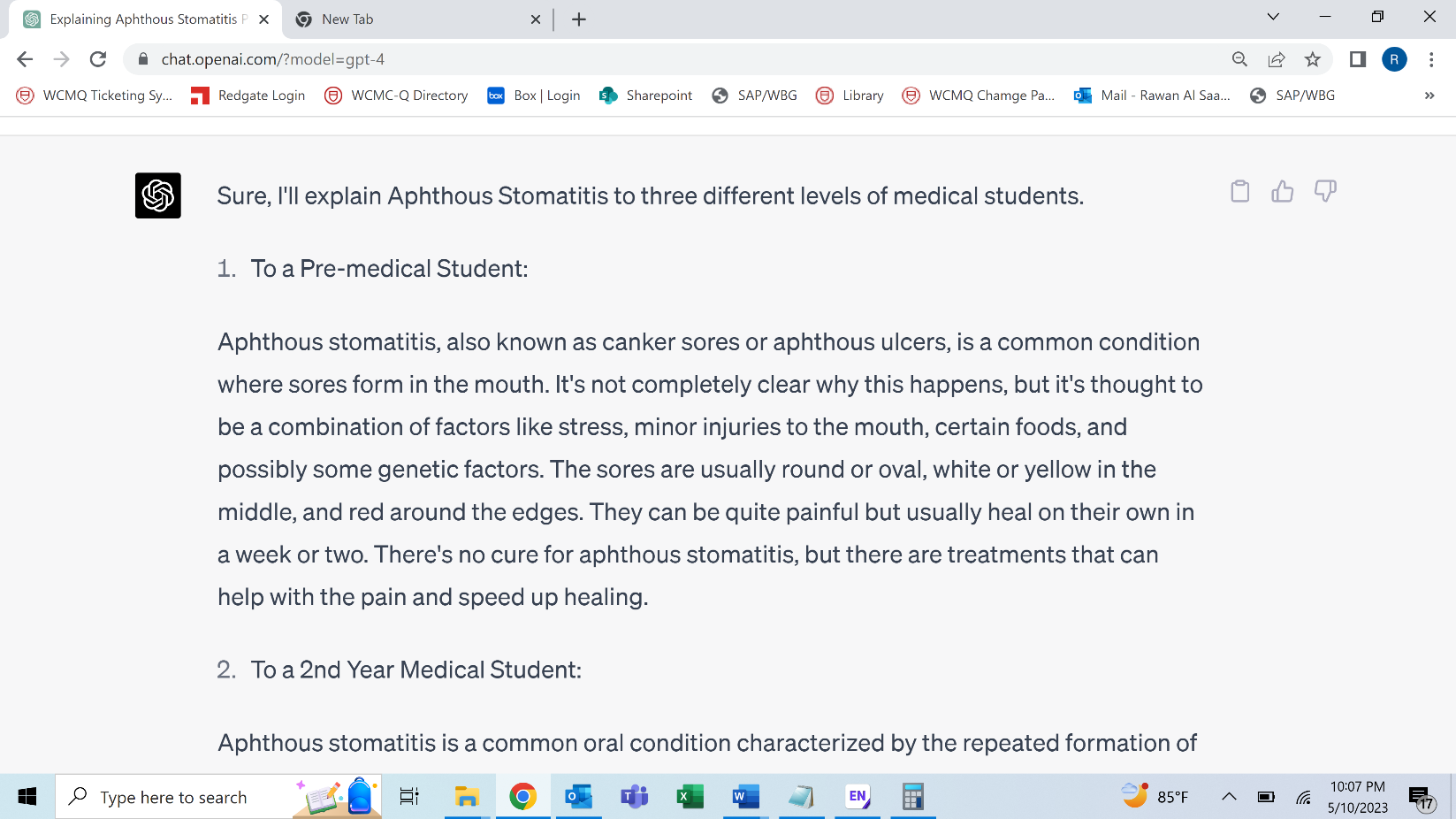


Figure S2: ChatGPT explanation of Aphthous Stomatitis to pre-medical student.


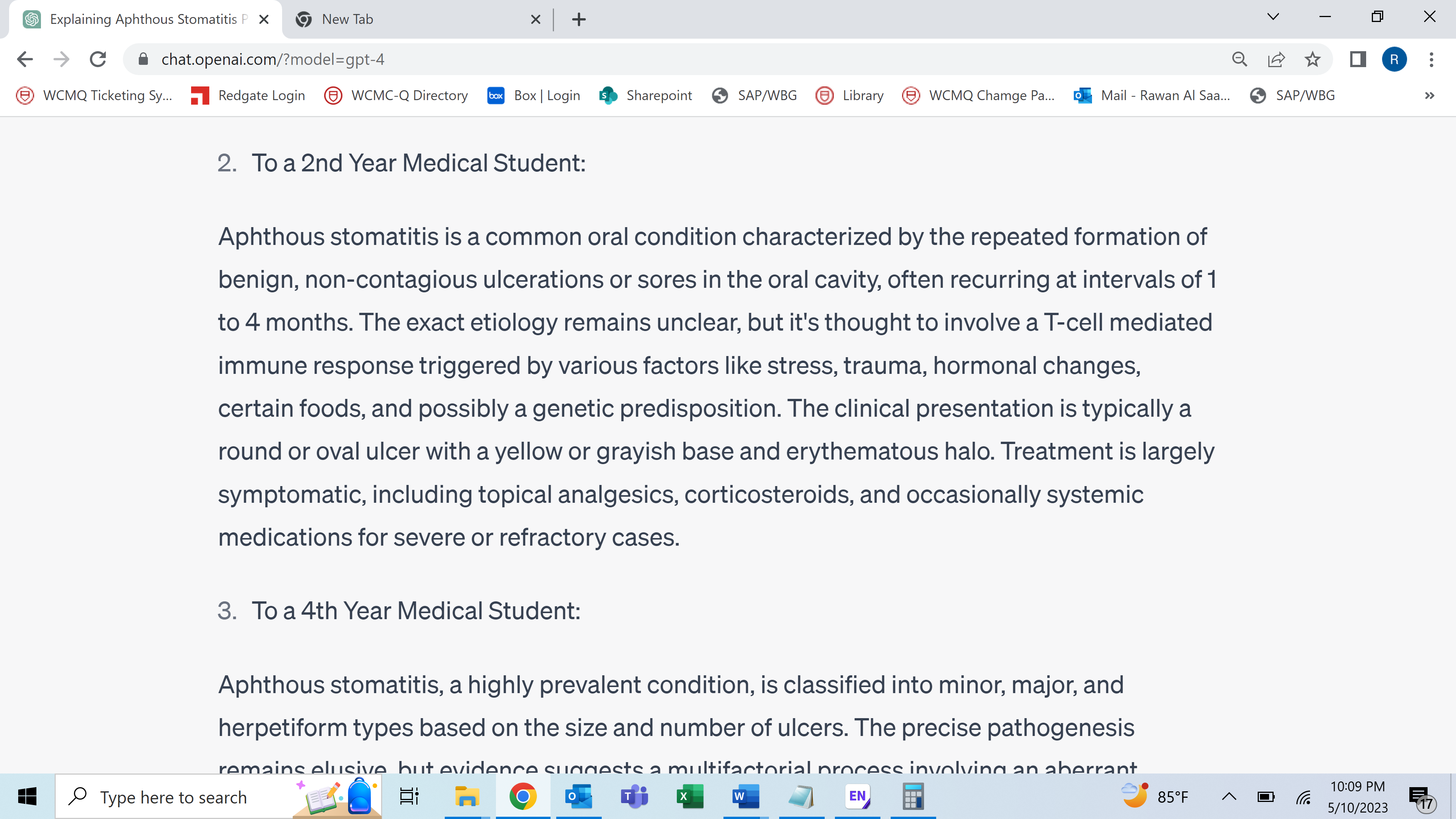


Figure S3: ChatGPT explanation of Aphthous Stomatitis to 2nd year medical student.


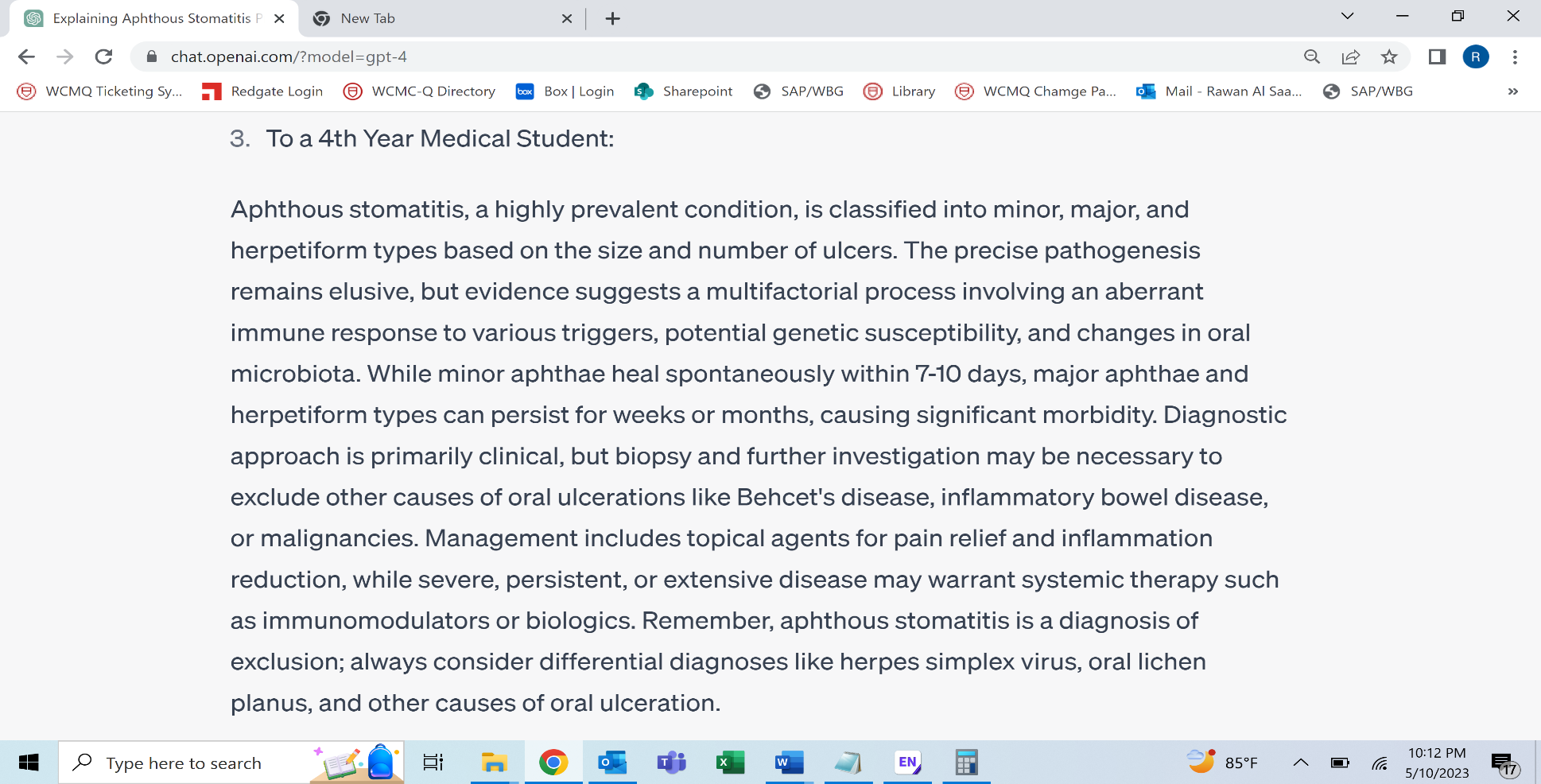


Figure S4: ChatGPT explanation of Aphthous Stomatitis to 4th year medical student.
