# Supplementary material for: Large Language Models in Medical Education: Opportunities, Challenges, and Future Directions
Source: JMIR Med Educ. 2023 Jun 1;9:e48291. doi: 10.2196/48291 (PMC10273039; doi:10.2196/48291)
Supplement: Multimedia Appendix 3 [file mededu_v9i1e48291_app3.docx]

Multimedia Appendix 3: Example of using LLMs (Petal) for documents analysis.


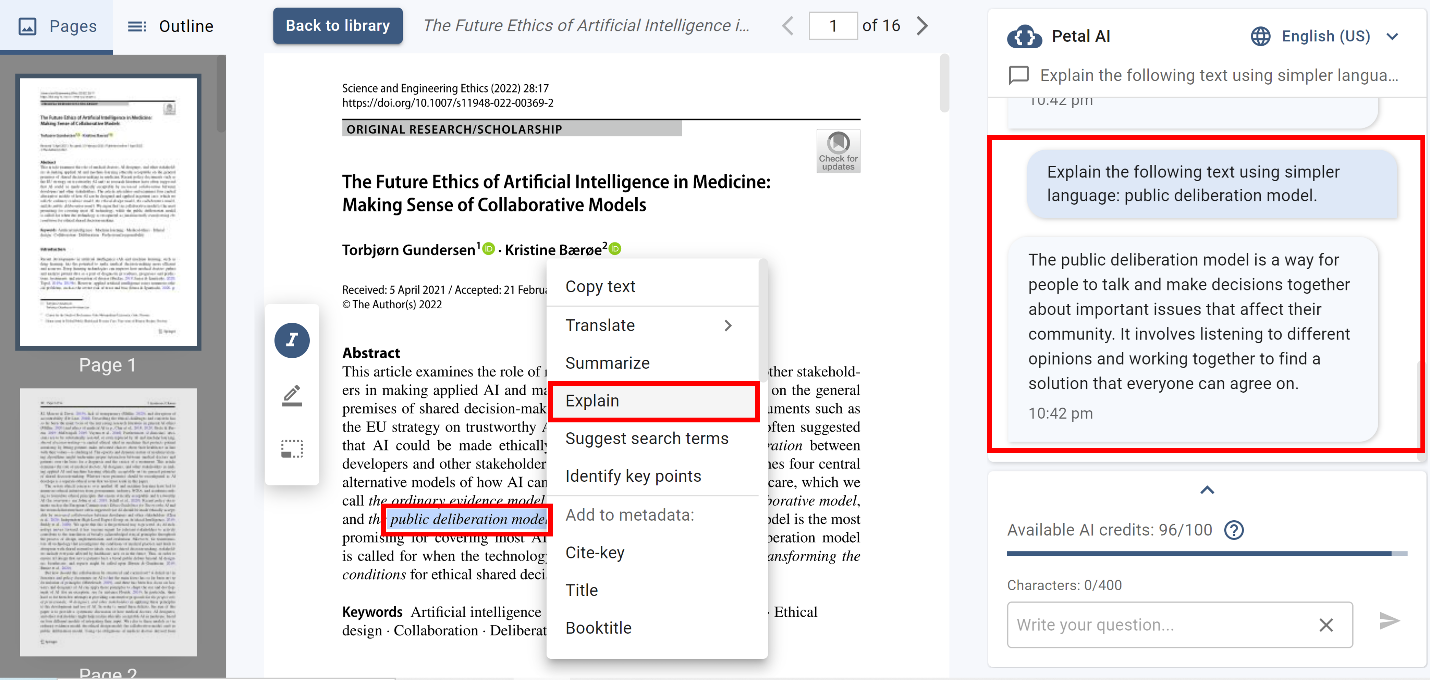


Figure S1: Using Petal to explain text in a research article.


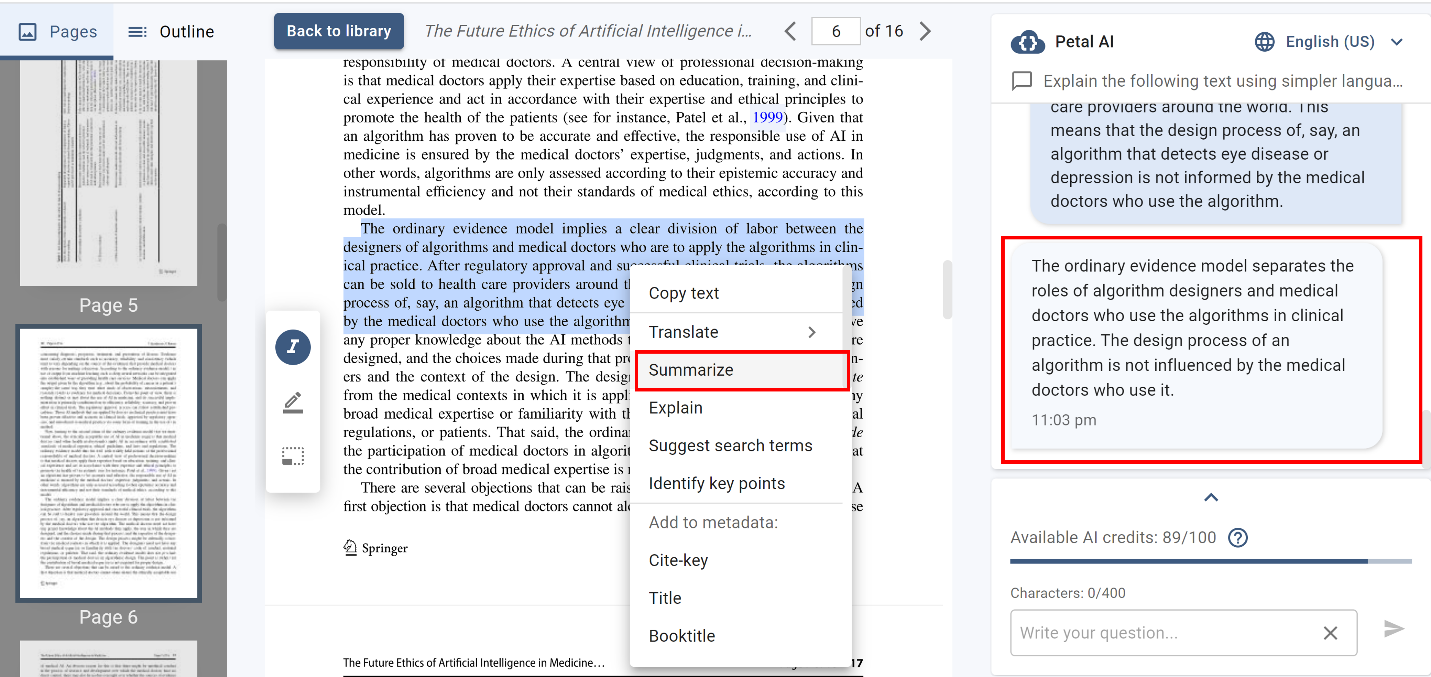


Figure S2: Using Petal to summarize text in a research article.
