# Supplementary material for: Large Language Models in Medical Education: Opportunities, Challenges, and Future Directions
Source: JMIR Med Educ. 2023 Jun 1;9:e48291. doi: 10.2196/48291 (PMC10273039; doi:10.2196/48291)
Supplement: Multimedia Appendix 4 [file mededu_v9i1e48291_app4.docx]

Multimedia Appendix 4: Example of using ChatGPT (GPT-4) to provide outline and references for research papers.


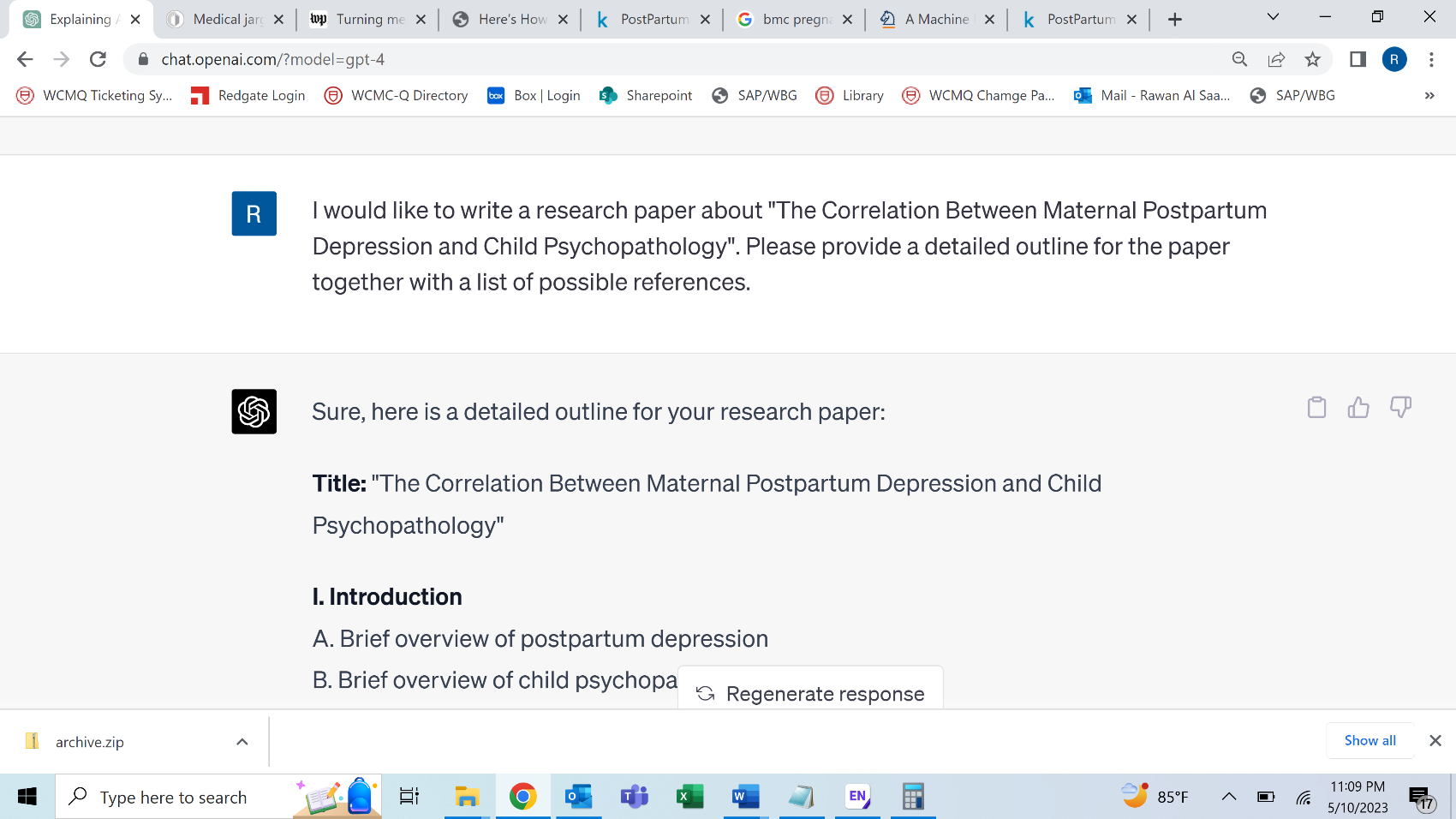


Figure S1: User prompt.


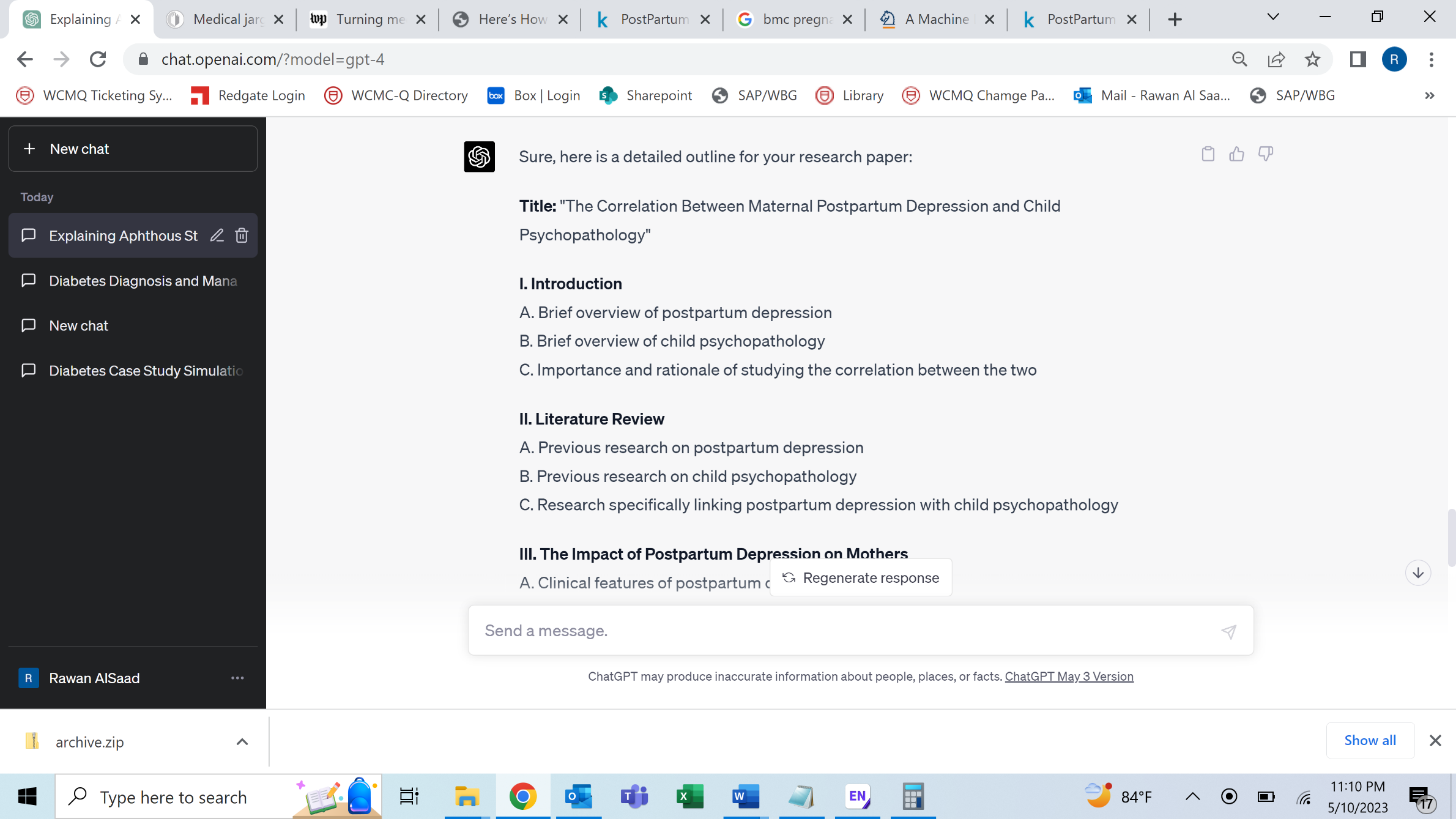


Figure S2: ChatGPT response.


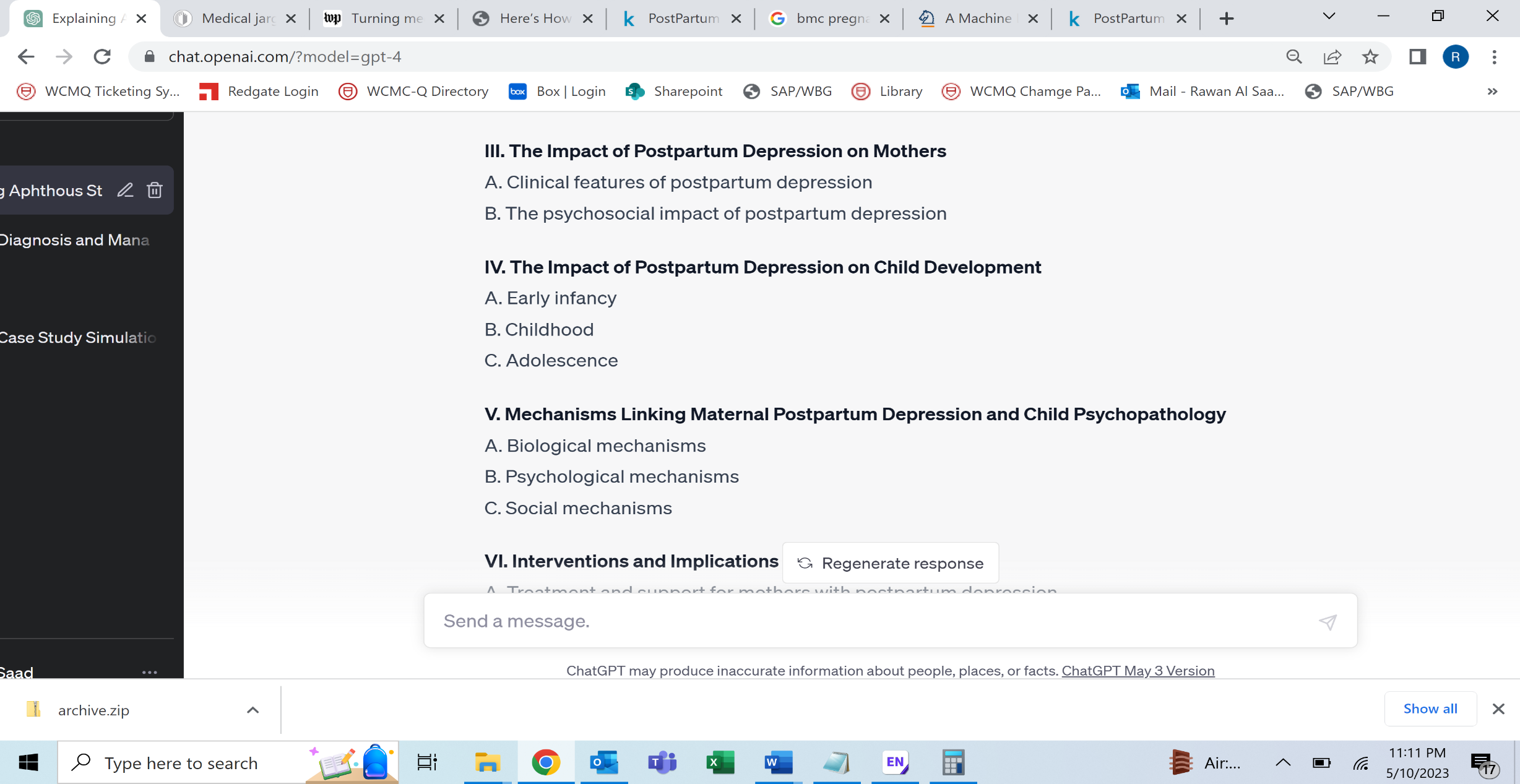


Figure S3: ChatGPT response (continued).


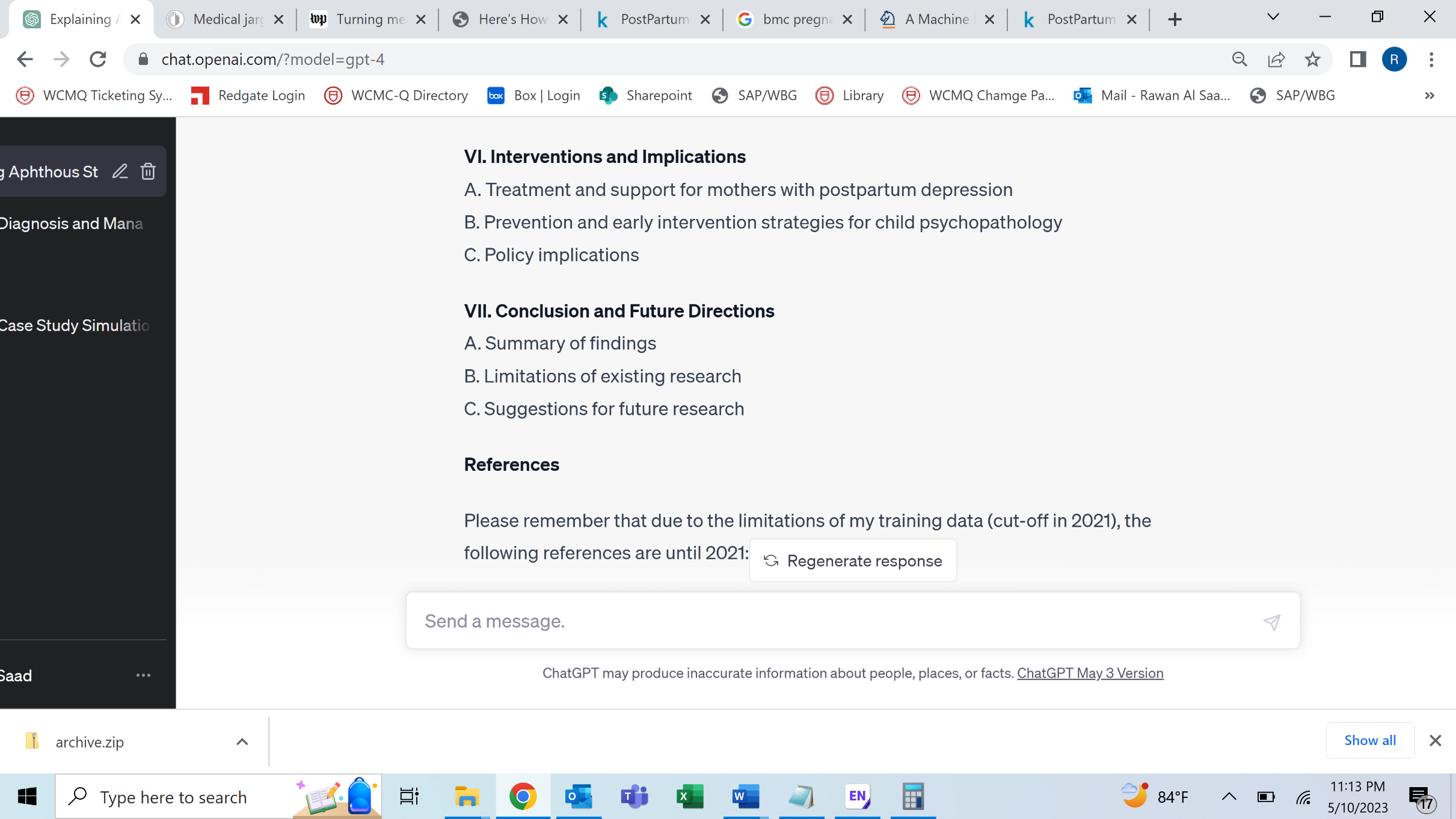


Figure S4: ChatGPT response (continued).


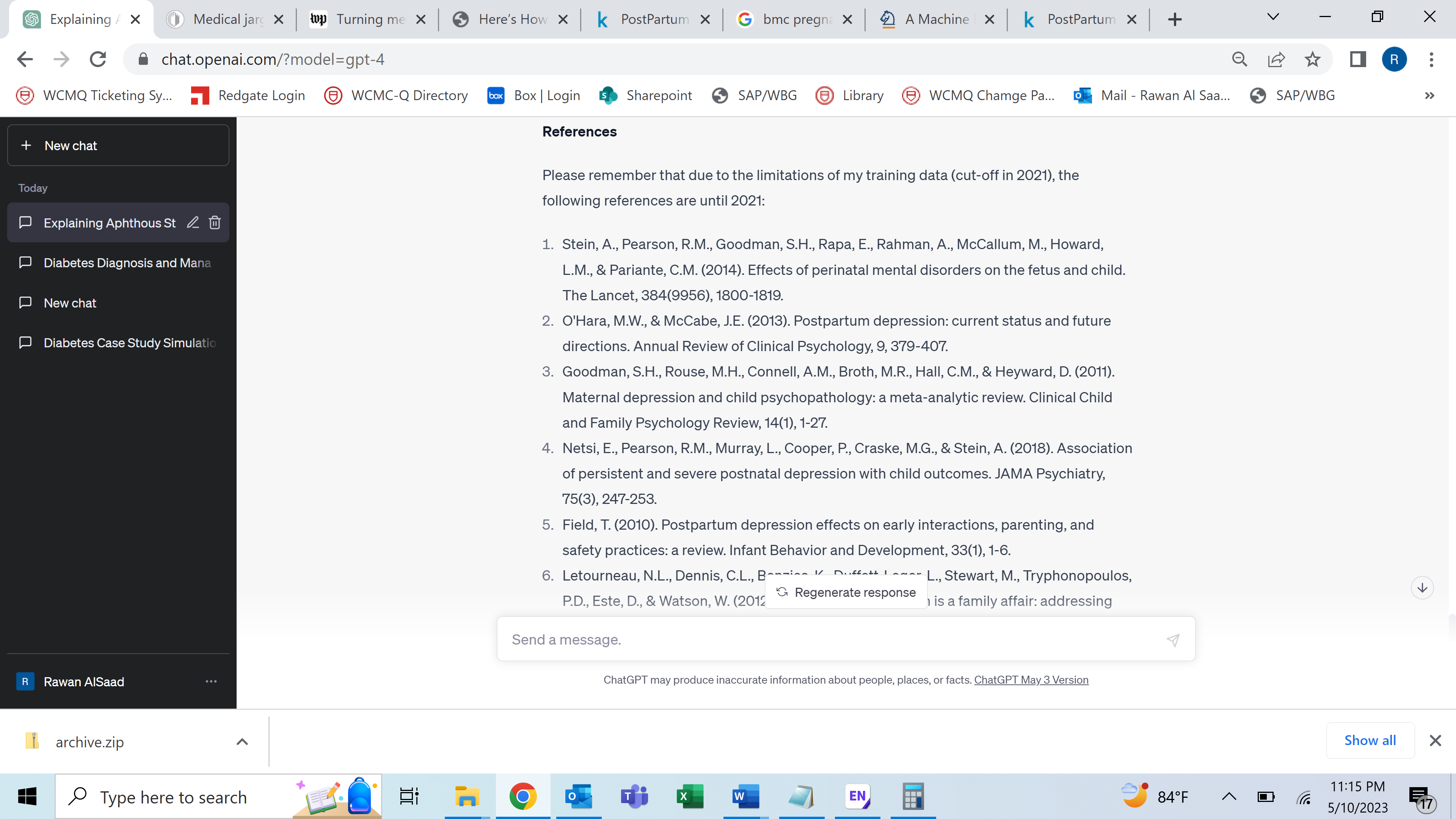


Figure S5: ChatGPT response (continued).
